# Advantages of Multiplexing Ability of the Orbitrap Mass Analyzer in the Multi-Mycotoxin Analysis

**DOI:** 10.3390/toxins15020134

**Published:** 2023-02-07

**Authors:** Dávid Rakk, József Kukolya, Biljana D. Škrbić, Csaba Vágvölgyi, Mónika Varga, András Szekeres

**Affiliations:** 1 Department of Microbiology, Faculty of Science and Informatics, University of Szeged, 52. Közép Fasor, 6726 Szeged, Hungary; 2 Research Group for Food Biotechnology, Institute of Food Science and Technology, Hungarian University of Agriculture and Life Sciences, 1022 Budapest, Hungary; 3 Faculty of Technology, University of Novi Sad, Bulevar cara Lazara 1, 21000 Novi Sad, Serbia

**Keywords:** fast mycotoxin measurement, parallel ion monitoring multiplexing, high throughput

## Abstract

In routine measurements, the length of the analysis time and nfumber of samples analysed during a time unit are crucial parameters, which are especially important for the food analysis, particularly in the case of mycotoxin determinations. High-resolution equipment, including time-of-flight or Orbitrap analyzators, can provide stable instrumental background for high-throughput analyses. In this report, a short, 1 min MS-based multi-mycotoxin method was developed with the application of a short column as a reduced chromatographic separation, taking advantages of the multiplexing and high-resolution capability of the QExactive Orbitrap MS possessing sub-1 ppm mass accuracy. The performance of the method was evaluated regarding selectivity, LOD, LOQ, linearity, matrix effect, and recovery, and compared to a UHPLC-MS/MS method. The final multiplexing method was able to quantify 11 mycotoxins in defined ranges (aflatoxins (corn, 2.8–600 μg/kg; wheat, 1.5–350 μg/kg), deoxynivalenol (corn, 640–9600 μg/kg; wheat, 128–3500 μg/kg), fumonisins (corn, 20–1500 μg/kg; wheat, 30–3500 μg/kg), HT-2 (corn, 64–5200 μg/kg; wheat, 61–3500 μg/kg), T-2 (corn, 10–800 μg/kg; wheat, 4–250 μg/kg), ochratoxin (corn, 4.7–600 μg/kg; wheat, 1–1000 μg/kg), zearalenone (corn, 64–4800 μg/kg; wheat, 4–500 μg/kg)) within one minute in corn and wheat matrices at the MRL levels stated by the European Union.

## 1. Introduction

Flow-injection analysis mass spectrometry (FIA-MS) methods by omitting or reducing chromatographic separation can provide high-throughput quantitative screening of analytes in less than 1 min and have been successfully applied for targeted quantitation in clinical, environmental, and forensic analysis using triple quadrupole instruments and high-resolution mass spectrometers, such as time-of-flight (TOF) and Orbitrap instruments [1]. However, simultaneous introduction of numerous compounds into the ion-source results in abundant matrix effects leading to sensitivity loss [2]. Recently, dilute-and-shoot methods [3] and the use of a short or guard column in the FIA-MS method (i.e., fast chromatography) [4] have been developed to overcome this effect.

The instruments with both quadrupole and Orbitrap analyzers (Q-Orbitrap) are the youngest member of the mass spectrometer’s family providing a number of scan modes, which are promising candidates for high-throughput analysis (HTA). The measurement modes resemble those found in conventional tandem MS instruments (MS/MS), including tandem quadrupole (QqQ) and quadrupole–time of flight (Q-TOF) mass filter couplings, but there are several characteristics that lead to significant differences among the QqQ, Q-TOF, and Q-Orbitrap scan modes. These differences are mainly the existence of both the C-trap and HCD cell, which are able to accumulate the ions and fragment them in a spatially separated way, respectively, as well as the absence of true scanning capability (only mathematically processed spectra available) [5]. Owing to the abovementioned technical implementations, the duty cycle and the applicability for the HTAs is mainly dependent on the measurement time within the Orbitrap core cell and limited by the ion storage capacity of the C-trap [5].

For targeted MS/MS analysis, certain types of acquisition methods are available, but to quantify a defined number of compounds, mainly the parallel reaction monitoring (PRM) mode is preferred, which is the product ion-scan acquisition of selected precursors with high resolution and high mass accuracy [6]. However, in PRM mode, the C-trap is idle during the main part of the duty cycle, which leads, on one hand, to the loss of a relevant part of the ion beam originating from the interface and, on other hand, to result in decreased data points. However, the need for HTAs led to the concept of multiplexing (MSX) in the case of Q-Orbitrap (QExactive) instruments, which manages the analysis of numerous simultaneously ionized compounds [7]. The analysis time for the Orbitrap mass analyzer is much longer than the time required for the collection of a sufficient amount of precursors in the C-trap, and the fragmentation of the precursors in the HCD-cell [5]. When 20 or more precursors are involved in the same scan cycle, the scan events are extended due to the long acquisition time for the Orbitrap mass analyzer, resulting in insufficient data points across a chromatographic peak for quantitative analysis. During an MSX acquisition, in one scan event, more than one precursor ion is transmitted by the quadrupole and collected by the C-trap, resulting in selected ion monitoring (SIM) MSX or optionally fragmented in the HCD cell leading to the PRM-MSX. Following the accumulation of the multiplexed ions, the precursors or fragments originating from different analyte precursors are injected into the Orbitrap mass analyzer in a single portion [5]. Therefore, the separated PRM cycles can involve only one MSX cycle, whose time is exactly the same as the time for a single PRM cycle under certain conditions. While the ions are detected, the ions are collecting for the next scan event; therefore, the performance of all mass analyzer components of the Orbitrap instrument is optimal during the course of an MSX scan event, resulting in fewer scan events and shorter scan cycles [5]. Depending on the type of instrument, 3 to 40 precursors with different *m/z* values can be monitored simultaneously in one scan event with the currently available Orbitrap instruments according to the manufacturer’s descriptions, meaning the possible MSX measurements of 3 to 40 analytes for SIM-MSX or 1 to 13 components with the monitoring of three *m/z* transitions (a target and two conforming ions) in the case of PRM-MSX.

Spectrum multiplexing capability of the Orbitrap has been applied mainly with data-independent analysis (DIA) [8,9], single ion monitoring (SIM), and parallel ion monitoring (PRM) [10] acquisition in LC-MS-based proteomics. In addition to peptide quantitation, multiplexed DIA target and non-target screening LC-MS methods have also been developed for determination of pesticides [11], veterinary drug residues [12], and mycotoxins [13].

Mycotoxins are low-molecular-weight secondary metabolites mainly produced by *Aspergillus*, *Penicillium*, and *Fusarium* species. They are highly noxious substances toward animals and humans, which cause serious problems in food and feed safety [14,15,16,17,18]. Therefore, regulations relating to mycotoxins have been established in many countries to protect the consumer from the harmful effects of these metabolites. Until 2003, approximately 100 countries covering over 80% of the world’s inhabitants had regulations or specialized guidelines for mycotoxins in food. The regulations were related to aflatoxins (AFs; AFB1, AFB2, AFG1, AFG2, and AFM1); trichothecenes (deoxynivalenol (DON), diacetoxyscirpenol, T-2 toxin (T2); and HT-2 toxin (HT2)); fumonisins (FB1, FB2, and FB3); agaric acid; ergot alkaloids; ochratoxin A (OTA); patulin; phomopsins; sterigmatocystin; and zearalenone (ZEA) [19]. Among them, five major mycotoxins of AFs: OTA; fumonisins; trichothecenes (DON, T2, and HT2); and ZEA, are of significant public health concern as they can cause adverse effects in humans and animals [20]. Furthermore, the European Union (EU) harmonized regulations for the maximum residue levels (MRL) of these mycotoxins in cereals including corn and wheat [21]. Analysis of these mycotoxins in foods and feeds are difficult due to their complex matrix constituents. Recent analytical solutions for mycotoxin analysis mainly include enzyme-linked immunosorbent assays (ELISA) [22], gas chromatography mass spectrometry (GC-MS) and GC-MS/MS [23,24], or high-performance liquid chromatography (HPLC) [25,26] coupled to various detectors. Among them, HPLC coupled to MS or MS/MS methods with high sensitivity, a low detection limit, and high-level confirmation reliability has become one of the most important analytical tools for multi-mycotoxin analysis [26,27,28,29,30]. The analysis time required for the most recent multi-mycotoxin methods is around 10 min [31,32]; however, routine laboratory applications always need faster methods to achieve higher productivity. Thus, mycotoxin analysis is an important consideration for HTA.

In this recent study, an FIA-MS multiplexed PRM (FIA-MSX-MS/MS) method requiring less than one minute is presented and compared to non-multiplexed PRM (UHPLC-MS/MS) acquisition for quantitative analysis of 11 mycotoxins in both corn and wheat matrices. Results provide an effective opportunity for an exceptionally high-throughput mycotoxin analysis. Based on our knowledge, this is the first report ever concerning an application of a quantitative PRM-MSX method.

## 2. Results and Discussion

### 2.1. Basic HRMS Conditions

Since electrospray ionization and consequently the sensitivity is strongly dependent on the solvent composition, different mobile phases were tested to obtain maximum mass spectrometric signal intensities. Zhou et al. reported that the response for [M+H]^+^ and [M+NH_4_]^+^ ions was found to be maximum around 60 and 80% methanol with proton and ammonium ion concentrations of approximately 10^−3^~10^−4^ M [33]. Based on these results, the optimization of the solvent composition was carried out with 70% methanol supplemented with formic acid, acetic acid, and ammonium formate at different concentrations. Using 0.5% formic acid resulted in the most abundant [M+H]^+^ signals for aflatoxins and fumonisins, while DON and ZEA formed the highest intensity protonated molecular ions at 0.3% formic acid concentration. Owing to the presence of the ammonium buffer in the mobile phase, ammonium adducts [M+NH_3_]^+^ produced the highest response for T2 and HT2. The signal intensity of OTA showed no remarkable differences within all solvent compositions. As a compromise, 0.5% formic acid and 5 mM ammonium formate were chosen as the mobile phase modifiers.

### 2.2. Establishment of the Multiplexing Method

Our goal was to develop a reliable high-throughput FIA-MS method for mycotoxin quantitation using multiplexed PRM acquisition. Because of their occurrence and toxicity, aflatoxins, OTA, fumonisins, trichothecenes (DON, T2 and HT2), and ZEA were selected as target compounds. For internal standard, VOL was applied. Although a short column was utilized in the FIA-MSX-MS/MS system, the matrix and the target compounds were not resolved properly; therefore, identification and quantitation could be based on the product ion (MS2) data. For each mycotoxin, one fragment ion was used for quantitation, while the identification was supported via two additional confirming fragment ions (first, second). Initially, to pick up the unique fragments, a priori fragmentation of all mycotoxins and the IS were carried out separately by injecting standard solutions at a concentration of 10 μg/mL. In addition to the exact mass, the optimal NCE for the detection of the three fragment ions was also established (Table 1).

Based on the instrumental background, the following criteria have to be considered to design a multiplexed PRM method:

(I) Spectrum multiplexing capability of the QExactive Plus instrument provides the accumulation of more than three precursors in one scan event. Owing to the PRM acquisition mode, all fragments of the isolated precursors are simultaneously detected; therefore, selection of the characteristic fragment ions of the precursors in the same scan event is mandatory for reliable qualitative and quantitative analysis.
(II) The target compounds are small molecular weight mycotoxins and some of them generate product ions with nearly the same *m/z* values. These compounds cannot participate in the same scan event.
(III) The minimum mass difference between two fragment ions in one scan event is determined to be 50 ppm.
(IV) As the narrowest isolation window of the quadrupole is 0.4 *m/z*, precursor ions with similar mass-to-charge ratio are filtered together. Consequently, these compounds have to be placed in the same scan event.
(V) Polarity switching is discarded during method development. Although polarity switching is relatively fast (around 500 ms in QExactive Plus instrument [34] compared to other HRMS instruments [35]), in polarity switching mode the positive and negative acquisition modes alternate; thus, the time of the polarity switches added to the total cycle time results in an excessive total cycle time, which prevents collection of the required data points for quantitation.

Considering these criteria, a PRM method applying a multiplexing degree of 4 was built using 4 scan events (Table 1). Fragmentation patterns of the mycotoxins revealed that the four aflatoxins could not be placed in the same event due to their similar fragments. Furthermore, the IS and the trichothecenes (DON, T2, HT2, and VOL) also have similar structural properties providing similar fragments, thus these molecules were also classified into different groups. However, VOL, HT2, and T2 could be placed in the same group with any of the aflatoxins. DON could only be placed in a group with AFB2. A specific fragment for ZEA was only found when it was grouped with HT2 and AFB1, forming the MSX group 3 (Table 1). OTA and both fumonisins could be grouped to any trichothecene–aflatoxin pair, thus these compounds were assigned to the 1, 2 and 4 MSX groups. Within the groups, the fragmentation energies of each compound were adjusted to different values to achieve the highest possible signal intensities (Table 1).

**Figure 1:**
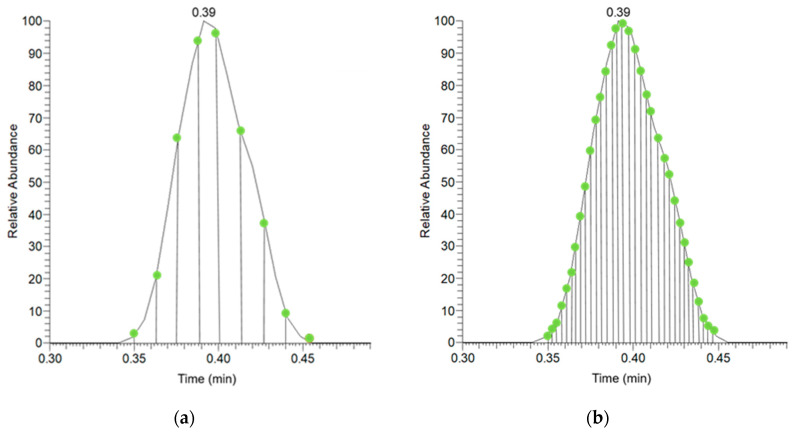
Data points of HT2 collected during the parallel measurement of the 12 compounds: (**a**) without the MSX technique (MS/MS); (**b**) applying the MSX technique (FIA-MSX-MS/MS).

The number of the data points within a peak has great importance regarding the detectability and sensitivity, as it is a general rule in the chromatography that at least 10 data points are needed to define the peak. The application of the multiplexing leads to the multiplication of data points, which is illustrated in Figure 1. The HT2 toxin could not be detected at the concentration of 40 ng/mL with the non-multiplexing technique due to the 9 data points collected, while the multiplexing method resulted in 33 data points (Figure 1). An increased number of data points within the peaks were observed for each mycotoxin using the FIA-MSX-MS/MS method.

In the final FIA-MSX-MS/MS method, compounds belonging to the same group are included in one measurement event, and the sum of the measurement events covers the entire measurement cycle. Resolution of 17,500 at *m/z* 200, recommended for PRM acquisition, was applied. This resolution requires a transient length (measurement time) of 64 ms. As all of the precursors were divided in four scan events, the total cycle time was 256 ms. In comparison, if each component tested was placed in a separate measurement event, the entire measurement cycle would have been exactly three times longer, 768 ms. This phenomenon is especially important for the case of mycotoxins that are difficult to detect. 

### 2.3. Validation of the FIA-MSX-MS/MS Method

The performance of the FIA-MSX-MS/MS method was evaluated regarding selectivity, LOD, LOQ, linearity, matrix effect, and recovery, and compared to a UHPLC-MS/MS method, a general non-multiplexing separation applying C18 stationary phase [36,37] and the same ion-source settings utilized for FIA-MSX-MS/MS. Although the same transitions of the mycotoxins were tested with the same fragmentation energy as in the case of the MSX method, only one precursor was analyzed at a time.

#### 2.3.1. Selectivity

To test the selectivity of the developed FIA-MSX-MS/MS method, the presence of any peaks in both corn and wheat blank matrix solutions were investigated confirming that there is no interfering signal for any mycotoxins. The possible false positive hits were defined as a signal response with a signal-to-noise ratio (S/N) larger than 3. Furthermore, each mycotoxin was injected into the system at high concentration level to ensure that the specific fragments could be derived only from their corresponding precursor.

#### 2.3.2. LOD, LOQ, and Linearity

To access sensitivity, LOD and LOQ values were determined using matrix blank extracts spiked with the internal standard and the mycotoxins in the concentration range 10,000–0.125 µg/kg. For each component, LOD and LOQ were defined as the concentration that could be detected with a S/N of 3 and 5, respectively, and were based on the weight of wheat and corn, referring to the complete analytical method, which includes the whole sample preparation (Table 2 and Table 3). Additionally, the LOQ values were considered as the lowest point in the calibration line.

To examine the limit parameters in corn, it can be concluded that the developed method completely fulfils the MRLs in this matrix. In the case of AFs, OTA, and HT2, the LOQ values were about half of the MRL, while the LOQs were remarkable lower than the limit value set in the European Union regulations for DON, ZEA, T2, and fumonisins (Table 2).

Although the MRLs are generally lower in wheat than in corn, the determined LOQs in this matrix were lower (Table 3). This can be explained by less fat and pigment contents in the wheat extracts compared to the corn extracts after QuEChERS sample preparation [38]. Fumonisins are not subject to the upper limit of protection for wheat, so the detections of these mycotoxins have not been investigated in this matrix. Overall, it can be concluded that in the cases for both corn and wheat matrices, each component can be reliably quantified at the MRL-level concentrations.

Comparing the multiplexing method with the UHPLC-MS/MS method, the sensitivity for aflatoxins, OTA, and ZEA in the case of corn matrix, aflatoxins and ZEA in wheat matrix were an order of magnitude better using detailed chromatographic separation. However, for the other mycotoxins, the LOD and LOQ values were at the same order of magnitude. Interestingly, the FIA-MSX-MS/MS method proved to be more sensitive for fumonisins.

With the application of the multiplex method, linear calibration curves were obtained for both wheat and corn matrices using internal standard calibration. The first point of the calibration lines was the LOQ value, while the upper limit of the quantitation (ULOQ) was the matrix-matched standard solution with the highest concentration that could still be properly fit to the curve. The correlation coefficient values for all compounds were greater than 0.99, thus meeting the relevant requirements of the general guidelines (Table 4). Using the UHPLC-MS/MS method, lower ULOQ values were obtained for the tested AFs, but at the same time the LOQ values were also lower, so both methods provide an identical linear dynamic range of two and a half orders of magnitude for these compounds. Fumonisins were only examined in a corn matrix, since the current legislation only applies to corn. FB1 and FB2 had a wider dynamic range when using the UHPLC-MS/MS method, similar to DON and ZEA. However, in the case of HT2, OTA, and T2 toxins, the dynamic range of the two methods was comparable.

#### 2.3.3. Evaluation of Matrix Effects

In LC-ESI-MS measurements, the response signal of the individual analytes can be greatly suppressed or enhanced in complex matrices due to the competition between the analyte and the matrix components in the ionization process. Matrix effect was investigated by comparing the peak area of the examined toxin in matrix blank extract spiked with the analyte to the peak area of the toxin in neat solution at the same concentration. The matrix factor was determined at two concentration levels, the first level was three times higher than the LOQ and the second level was 80% of the ULOQ. 

Ion enhancement was present only for OTA in corn and ZEA in wheat, while ion suppression was observed for all other mycotoxins in both matrices. Matrix effect has been classified as soft (±20% signal suppression or enhancement), medium (±20–50%), and strong (more than 50% and less than −50%) [39]. Soft matrix effects were observed for OTA and FB2 (Table 5). Only FB1 and ZEA exhibited medium signal suppression or enhancement. AFs and DON were found to have the most pronounced signal suppression. This result is in agreement with the observation by Bonfiglio et al. demonstrating that ESI response suppression is compound dependent—suppression was greatest with the most polar analyte [40]. Furthermore, matrix effect was dependent on the nature of the matrix for all mycotoxins, except for AFB2, AFG1, and DON. In the case of HT2, T2, and ZEA toxins, the corn matrix has a more remarkable signal-intensity-reducing effect than the wheat matrix (Table 5). Similar results were reported by Fernandes et al. [41]. The target components can be determined in individual samples with sufficient accuracy if the matrix effect is approximately constant in the entire tested concentration range. The generally applied acceptance limit is an RSD value of 20% for the matrix factor, which was completed by our FIA-MSX-MS/MS method with the highest value of 19.9% in the case of AFG2 in corn matrix.

The high matrix effect can be explained by the QuEChERS sample preparation technique and the lack of the proper chromatographic separation. Using QuEChERS clean-up for quantification of mycotoxins in maize, a remarkable signal suppression/enhancement was also observed by other researchers [41,42]. Matrix effect can be significantly reduced by using other sample preparation methods or combining them [43]. Woo et al., comparing solid phase extraction (SPE), QuEChERS, and immunoaffinity clean up (IAC), observed better performance using IAC; however, the procedure has higher costs and is more time-consuming [44]. Comparing matrix factor values determined for the FIA-MSX-MS/MS and UHPLC-MS/MS methods, the pronounced signal reduction detected in our FIA experiments can be due to the absence of detailed chromatographic separation (Table 5).

#### 2.3.4. Recovery Studies

The accuracy and the precision of the developed method were established through recovery experiments including the whole sample preparation and the FIA-MSX-MS/MS method. The recovery tests were conducted by spiking blank corn and wheat samples at levels corresponding to 80 and 120% of the MRL for each mycotoxin. Six replicates were performed for both concentrations. Apparent recoveries [45], a combination of the extraction efficiency and matrix effect, were determined due to the strong signal suppression for most of the analytes. The apparent recovery was calculated by comparing the measured concentration using the matrix-matched calibration curves to the spiked concentration of each mycotoxin (Table 6).

The recovery values ranged from 0.71 to 0.93 in corn and from 0.71 to 0.87 in wheat with associated RSD lower than 16.5%. All recoveries comply with the eligibility criteria set out in Regulation 401/2006/EC [46] of the European Commission. Similar recovery values and similar variability among recoveries were established using the UHPLC-MS/MS method (Table 6).

During routine measurement the LOQ is modified by the recovery value [46]. Performing this adjustment, the corrected LOQ values (Table 3) were more than the corresponding MRLs.

Finally, it can be concluded that the recovery and repeatability of the entire analytical procedure including sample clean-up and the detection complies with the general analytical requirements. It is important to note that the LOD and LOQ values are based on the weight of wheat and maize, respectively, and refer to the complete analytical method, which contains the sample preparation, including the 10-fold concentration step. This concentration step could likely be omitted in the case of recent or later quadrupole-Orbitrap hybrid instruments marketed after the QExactive Plus series.

## 3. Conclusions

As well known, there are numerous challenges in mycotoxin analysis, including the complexity of the matrices, the heterogeneities of the analytes, and the usually complex sample processing; the MRLs are remarkably low and multiple mycotoxins can be presented in the same sample. The aim of our research was to develop a quick, high-throughput MS-based multi-mycotoxin method taking advantage of the multiplexing opportunity and high-resolution ability of the QExactive Orbitrap MS. After assessing the basic principles necessary for successful development of a PRM-MSX method, we established an FIA-MSX-MS/MS method for quantitation of 11 mycotoxins. A short column was utilized to provide high-throughput analysis. The performance of the method was evaluated in corn and wheat matrices regarding selectivity, LOD, LOQ, linearity, matrix effect, and recovery, and compared to a UHPLC-MS/MS method. The developed method is suitable for high-throughput quantitative screening of 11 mycotoxins in corn and wheat matrices within one minute.

## 4. Materials and Methods

### 4.1. Chemicals

Mycotoxin standards including AFB1, AFB2, AFG1 AFG2, DON, HT2, OTA, T2, VOL, and ZEA were purchased from Merck Ltd. (Budapest, Hungary), and FB1 and FB2 from Fumizol Ltd. (Szeged, Hungary). The MgSO_4_, NaCl, trisodium citrate, and ammonium formate, along with the LC-MS grade acetic acid and formic acid, were also from Merck Ltd. (Budapest, Hungary), while applied LC-MS grade solvents (H_2_O, methanol–MeOH, acetonitrile–MeCN) were obtained from VWR Ltd. (Debrecen, Hungary). Primary/secondary amin bulk SPE absorbent (PSA) and bulk C18 silica-gel were purchased from Merck Ltd. (Budapest, Hungary).

### 4.2. Preparation of Standard Solutions and Validation Samples

The individual stock solutions of mycotoxins (AFB1, AFB2, AFG1 AFG2, DON, FB1, FB2, HT2, OTA, T2, and ZEA) were prepared by diluting 2 mg of each mycotoxin in 1 mL matrix solution. The extraction solution was MeCN/H_2_O/acetic acid (79/20/1, *v*/*v*/*v*%) containing the internal standard (VOL) in 50 ng/mL concentration.

For preparing intermediate solutions, the individual stock standard solutions were diluted in the matrix solution, and then five level toxin–mixture solutions in matrix solution were created with the following concentrations: 100,000; 10,000; 1000; 100; and 10 ng/mL. The working solutions were prepared from these solutions at 16 levels in the range 50,000–0.5 ng/mL with the matrix solution. The matrix-matched calibration levels were also prepared at 16 levels in the concentration range 2500–0.025 ng/mL with the mixing of 180 µL matrix solution and 10 µL working solution, which are equal according to the applied sample preparation with the concentration levels 10,000–0.1 µg/kg and 12,500–0.125 µg/kg in the case of corn and wheat, respectively. All these solutions were stored at −20 °C.

For the determination of matrix effects, samples were prepared at two concentration levels (MEC1 and MEC2) for each mycotoxin in six repetitions, both in MeCN/H_2_O/acetic acid (79/20/1, *v*/*v*/*v*%) containing the internal standard (VOL) in 500 ng/mL concentration and in matrix solution (190 µL matrix solution, 10 µL mycotoxin working solution). In the case of samples (corn and wheat) used for matrix-effect determination, all examined mycotoxins were found to be below the LODs. The MEC1 and MEC2 were specified for each mycotoxin as twice the limit of quantitation (LOQ) and 80% of the upper limit of quantitation (ULOQ), respectively. The concentrations of MEC1 and MEC2 according to the applied sample preparation were for corn and wheat in the case of FIA-MSX-MS/MS method (mycotoxin MEC1 in corn, MEC2 in corn, MEC1 in wheat, MEC2 in wheat): AFB1 (5.6 µg/kg, 480 µg/mL, 3 µg/kg, 280 µg/kg), AFB2 (4.8 µg/kg, 480 µg/mL, 2 µg/kg, 280 µg/kg), AFG1 (5.6 µg/kg, 480 µg/mL, 3 µg/kg, 280 µg/kg), AFG2 (5.6 µg/kg, 480 µg/mL, 3 µg/kg, 280 µg/kg), DON (1280 µg/kg, 7680 µg/mL, 960 µg/kg, 1920 µg/kg), FB1 (40 µg/kg, 960 µg/kg not determined in wheat matrix), FB2 (40 µg/kg, 1200 µg/kg, not determined in wheat matrix), HT2 (128 µg/kg, 4160 µg/mL, 40 µg/kg, 280 µg/kg), OTA (7 µg/kg, 480 µg/mL, 4 µg/kg, 280 µg/kg), T2 (20 µg/kg, 640 µg/mL, 30 µg/kg, 480 µg/kg), and ZEA (128 µg/kg, 3840 µg/mL, 120 µg/kg, 480 µg/kg). According to the applied sample preparation, the MEC1 and MEC2 concentrations were the following in the two examined matrices, in the case of UHPLC-MS/MS method (mycotoxin MEC1 in corn, MEC2 in corn, MEC1 in wheat, MEC2 in wheat): AFB1 (5.6 µg/kg, 480 µg/mL, 3 µg/kg, 280 µg/kg), AFB2 (4.8 µg/kg, 480 µg/mL, 2 µg/kg, 280 µg/kg), AFG1 (5.6 µg/kg, 480 µg/mL, 3 µg/kg, 280 µg/kg), AFG2 (5.6 µg/kg, 480 µg/mL, 3 µg/kg, 280 µg/kg), DON (1280 µg/kg, 7680 µg/mL, 960 µg/kg, 1920 µg/kg), FB1 (40 µg/kg, 960 µg/kg not determined in wheat matrix), FB2 (40 µg/kg, 1200 µg/kg, not determined in wheat matrix), HT2 (128 µg/kg, 4160 µg/mL, 40 µg/kg, 280 µg/kg), OTA (2 µg/kg, 800 µg/kg, 16 µg/kg, 1120 µg/kg), T2 (8 µg/kg, 200 µg/kg, 16 µg/kg, 1120 µg/kg), and ZEA (8 µg/kg, 400 µg/kg, 5 µg/kg, 192 µg/kg). For recovery studies, the samples were prepared in two concentrations (RC1 and RC2) and in six repetitions. During the procedure, 1 g powdered blank corn or wheat samples were spiked with 100 µL mycotoxin mixture and let to dry in dark for 16 h. Then, the described sample preparations were applied. The concentrations of the fortified corn samples were 80 and 120% of the MRL: AFB1 (4 µg/kg, 6 µg/kg), AFB2 (4 µg/kg, 6 µg/kg), AFG1 (4 µg/kg, 6 µg/kg), AFG2 (4 µg/kg, 6 µg/kg), DON (1400 µg/kg, 2100 µg/kg), FB1 (800 µg/kg, 1200 µg/kg), FB2 (800 µg/kg, 1200 µg/kg), HT2 (80 µg/kg, 120 µg/kg), OTA (4 µg/kg, 6 µg/kg), T2 (80 µg/kg, 120 µg/kg), and ZEA (160 µg/kg, 240 µg/kg), for RC1 and RC2, respectively. The concentrations of the fortified wheat samples were AFB1 (1.6 µg/kg, 2.4 µg/kg), AFB2 (1.6 µg/kg, 2.4 µg/kg), AFG1 (1.6 µg/kg, 2.4 µg/kg), AFG2 (1.6 µg/kg, 2.4 µg/kg), DON (1000 µg/kg, 1500 µg/kg), HT2 (40 µg/kg, 60 µg/kg), OTA (4 µg/kg, 6 µg/kg), T2 (40 µg/kg, 60 µg/kg), and ZEA (80 µg/kg, 120 µg/kg), for RC1 and RC2, respectively.

### 4.3. Sample Preparations

Corn samples were ground using a Laboratory Mill 3310 (Perkin Elmer, Waltham, MA, USA) with its rotating disc at position 0 and 1.00 ± 0.01 g was weighed into 15 mL test tubes and extracted for 90 min with a Heidolph overhead shaker (Schwabach, Germany) with 4 mL of MeCN/H_2_O/acetic acid (79/20/1, *v*/*v*/*v*%) containing the internal standard (VOL) at 50 ng/mL concentration. Then, a modified QuEChERS method was applied for the clean-up of the mycotoxins according to Zhang et al. [47] with minor modifications. The samples were centrifuged at 8200× *g* for 10 min (Eppendorf R, Hamburg, Germany) and a 3 mL supernatant was transferred into new 15 mL tubes containing 0.8 g anhydrous MgSO_4_, 0.2 g NaCl, and 0.2 g trisodium citrate. The tubes were shaken by hand for 1 min immediately and vortexed for 2 min (VWR Ltd., Debrecen, Hungary). Then, the tubes were centrifuged for another 15 min at 8200× *g* (Eppendorf, Hamburg, Germany). A total of 2.5 mL of extract was transferred to a new 15 mL tube containing 200 mg C18 and 200 mg PSA, followed by vigorous shaking (2 min) and centrifugation (15 min at 8200× *g*). Then, 2 mL of the cleaned-up extracts was evaporated under a gentle stream of nitrogen at 40 °C and its volume was set to 0.2 mL with MeCN/H_2_O/acetic acid (79/20/1, *v*/*v*/*v*%) solution, vortexed for 30 s, sonicated for 4 min and, finally, passed through a 0.2 μm PTFE filter (Whatman, Buckinghamshire, UK). Wheat samples were also ground using a Laboratory Mill 3310 (Perkin Elmer, Waltham, MA, USA) with its rotating disc at position 0 and 1.00 ± 0.01 g blank wheat sample weighed into test tubes and extracted with 5 mL extraction solvent (MeCN/H_2_O/acetic acid (79/20/1, *v*/*v*/*v*%) containing the internal standard (VOL) in 50 ng/mL concentration using an overhead shaker for 90 min. The further sample preparation steps were the same as applied for corn samples. These sample preparation procedures were also applied to obtain the matrix solutions for the matrix matched calibrations. The entire sample preparation required 180 min, including the extraction (90 min) and QuEChERS clean-up procedure (50 min), together with evaporation, sonication, and filtration (40 min).

### 4.4. Instruments and Analytical Parameters

#### 4.4.1. General Instrumental Parameters

The UHPLC-HRMS system consisted of a Dionex UltiMate 3000 UHPLC coupled to a Thermo Scientific Q-Exactive Plus Orbitrap mass spectrometer (Thermo Scientific, San Jose, CA, USA) operating with a heated electrospray ionization (HESI II) source in positive ionization mode. For mass calibration of the instrument, Pierce™ LTQ Velos ESI Positive Ion Calibration Solution (Thermo Fisher Scientific, Waltham, MA, USA) was used.

During the HRMS optimization and in both FIA-MSX-MS/MS and UHPLC-MS/MS methods, the ion-source parameters were spray voltage 4 kV; sheath gas (N_2_) 50; auxiliary gas (N_2_) 20; sweep gas (N_2_) 1; capillary temperature 300 °C; S-lens RF level 40; and auxiliary gas heater temperature 400 °C. Value for automatic gain control (AGC) target was set at 1 × 10^6^, with a resolution of 35,000 FWHM (full width at half maximum, *m/z* = 200). The accumulation time limit of the ions per scan event was 10 ms.

The instrument control and the data processing were performed using QExactive Plus Tune 2.8 and TraceFinder General Quan 4.1 software (Thermo Fisher Scientific, Waltham, MA, USA), while Xcalibur software v. 4.0 (Thermo Fisher Scientific, Waltham, MA, USA) was applied for the spectral examinations.

#### 4.4.2. Optimization of Mass Spectrometric Conditions

The HRMS optimization was performed using FIA without a chromatographic column, injecting 5 µL of each mycotoxin standard (1 µg/mL in MeCN/H_2_O/acetic acid (79/20/1, *v*/*v*/*v*%) solution) at a flow rate of 500 µL/min. In full MS scan mode, precursor ion *m/z* values of the examined mycotoxins were identified and selected. To reach the highest signal for the precursors, the following eluents were investigated: (C1) H_2_O:MeOH (3:7) + 5 mM ammonium formate and 0.1% formic acid; (C2) H_2_O:MeOH (3:7) + 5 mM ammonium formate and 0.3% formic acid; (C3) H_2_O:MeOH (3:7) + 5 mM ammonium formate and 0.5% formic acid; (C4) H_2_O:MeOH (3:7) + 5 mM ammonium formate and 0.3% acetic acid; (C5) H_2_O:MeOH (3:7) + 0.3% acetic acid; (C6) H_2_O:MeOH (3:7) + 0.3% formic acid; (C7) H_2_O:MeCN (3:7) + 0.3% formic acid; (C8) H_2_O:MeOH (3:7); and (C9) H_2_O:MeCN (3:7). To find the optimal fragmentation parameters in PRM mode, the fragmentation energies were incremented stepwise with 10 units within the range 30–80 NCE using the optimized eluent composition (C3).

#### 4.4.3. Development of the Rapid FIA-MSX-MS/MS Method

All analyses were performed at the same mass spectrometric conditions as mentioned in the Section 4.4.2. However, to focus the analytes chromatographically, a Hypersil Gold C18 (Thermo Fisher Scientific, Waltham, MA, USA) 50 × 2.1 mm, 1.9 μm column was applied, while the isocratic mobile phase was the eluent composition C3, constituted of H_2_O:MeOH (3:7) with 5 Mm ammonium formate and 0.5% formic acid with a flow rate of 500 µL/min. The column oven temperature was 30 °C and the injection volume was 5 µL. The mass spectra were acquired with PRM mode and the accumulation time limit of the ions per scan event was 10 ms. The MSX count was set to 4, while the isolation width of the quadrupole was set to 0.4 *m/z*. All detectable fragments of the investigated mycotoxins were collected and listed, using individual mycotoxin samples with 10 µg/mL concentration, dissolved in MeCN/H_2_O/acetic acid (79/20/1, *v*/*v*/*v*%) solution. Altogether, twelve compounds were grouped in four MSX scan events. The principles and the concept of creation of the multiplexed method are detailed in the Results section. Identification of the compounds was based on the detection of three fragment ions (1 target and 2 confirming ions) with a mass accuracy of 3 ppm (Table 1). In addition, the ratio of the target and the confirming ions was calculated and compared to the ratio obtained for standards, where 20% maximum deviations were allowed. The total run time for the method was 1 min.

#### 4.4.4. UHPLC-MS/MS Method

The method applied was based on the work of Kaczynski et al. and Jia et al. [13,48]. For the separation, the previously applied Hypersil Gold C18 (Thermo Fisher Scientific, Waltham, MA, USA) 50 × 2.1 mm, 1.9 μm column was used, which was thermostated at the same temperature as for the FIA-MSX-MS/MS measurements, while the mobile phase was constituted of H_2_O with 5 Mm ammonium formate (A) and MeOH with 5 Mm ammonium formate and 0.5% formic acid (B). The mobile phase flow rate was 500 µL/min and the gradient program started with eluent B at 5% for 3 min, changing to 74% until 14.5 min, which was increased to 95% until 15.0 min with the value being held for two minutes and then decreased to the initial 5% in 0.1 min and kept constant for 1.9 min, resulting in a run of 19 min in total. The injection volume was 5 µL. The mass spectrometric data were acquired in PRM mode with the 0.4 *m/z* quadrupole isolation window using the same transitions as in the case of FIA-MSX-MS/MS (Table 1), but without any multiplexing.

### 4.5. Validation Parameters

#### 4.5.1. Selectivity

To test the selectivity of the FIA-MSX-MS/MS and UHPLC-MS/MS methods, the presence of any peaks in both the extraction solvent and the blank matrix solution were investigated, confirming that there were no interfering signals for all mycotoxins. The possible false positive hits were defined as an apparent signal response with S/N higher than 3. Furthermore, each mycotoxin was injected into the system at a high concentration level (10 µg/mL) to ensure that the specific fragments of each precursor could be detected only from their run and the fragments of other mycotoxins or other interfering peaks could not be detected.

#### 4.5.2. LOD, LOQ, and Linearity

Sensitivity was evaluated by limit of detection (LOD) and limit of quantitation (LOQ) values. The LODs and LOQs were evaluated by analyzing matrix-matched samples and were determined in a signal-to-noise ratio ≥3 (LOD) and ≥5 (LOQ), with respect to the confirming peak. When necessary, intermediate calibration levels were diluted.

Linearity was evaluated for each mycotoxin using the matrix-matched calibration curve of each standard at different concentration levels, starting from the LOQ for all analysed mycotoxins. The calibration curves were considered linear until the correlation coefficient (R^2^) value was higher than 0.99 and the accuracy of the concentration levels remained under 15% except for the LOQ, where 20% was allowed. The upper concentration values of the linear range were expressed as the upper limit of quantitation (ULOQ). The LOD, LOQ, and linearity were calculated from six independent solution preparations for both UHPLC- MS/MS and FIA-MSX-MS/MS measurements.

#### 4.5.3. Evaluation of Matrix Effects

To assess the possible matrix effect on the mass spectrometric response (signal enhancement or suppression), the matrix factor was investigated by calculating the ratio between the peak area of each mycotoxin in the matrix solvent and in the extraction solvent at two concentration levels (MEC1 and MEC2) [49]. Each concentration level was prepared in six independent repetitions and after the runs, the matrix factors were determined for all sample pairs and their averages and the related RSD% were calculated. Negative results were obtained when signal suppression occurs, while positive results corresponded to signal enhancement due to matrix effects.

#### 4.5.4. Recovery Studies

The accuracy of the entire FIA-MSX-MS/MS method coupling to the applied sample preparation was evaluated with the recovery test as the ratio of the measured concentration and the known spiked concentration in both corn and wheat samples at two concentration levels (RC1 and RC2, 80 and 120% of the MRL), and was expressed as [(measured concentration)/(added concentration)] × 100. For the quantitation, a matrix-matched calibration was applied. The evaluated recovery percentages of the repetitions for both concentration levels were averaged for each mycotoxin (n = 12 per mycotoxin) and the RSD% was determined [36].

## Figures and Tables

**Table 1:** Q-Orbitrap parameters for the analyzed 11 mycotoxins and the IS compound.

Mycotoxin	Abbrev.	Precursor Ion	Theoretical Mass (*m/z*)	Target Ion Mass (*m/z*)	1st/2nd Confirming Ion Masses (*m/z*)	NCE ^1^	MSX Group ^2^
Aflatoxin B1	AFB1	[M+H]^+^	313.0707	241.0488	269.0436/242.0566	60	3
Aflatoxin B2	AFB2	[M+H]^+^	315.0863	259.0593	287.0909/243.0645	60	4
Aflatoxin G1	AFG1	[M+H]^+^	329.0656	215.0694	200.0461/214.0617	60	1
Aflatoxin G2	AFG2	[M+H]^+^	331.0812	245.0799	217.0851/189.0541	60	2
Deoxynivalenol	DON	[M+H]^+^	297.1333	175.0752	105.0706/79.0545	50	4
Fumonizin B1	FB1	[M+H]^+^	722.3958	334.3111	95.0861/74.0602	40	4
Fumonizin B2	FB2	[M+H]^+^	706.4008	336.3269	318.3164/159.029	40	1
HT2 toxin	HT2	[M+NH_4_]^+^	442.2435	169.1010	157.1012/197.0956	40	3
Ochratoxin	OTA	[M+H]^+^	404.0895	257.0209	358.0843/239.0102	20	2
T2 toxin	T2	[M+NH_4_]^+^	484.2541	169.1008	154.0776/215.1061	50	2
Verrucarol (IS)	VOL	[M+H]^+^	267.1591	185.1321	157.1012/175.1115	50	1
Zearalenone	ZEA	[M+H]^+^	319.1540	69.0700	205.0854/283.1326	40	3

^1^ NCE—normalized collision energy applied in the fragmentation; ^2^ MSX group—multiplexing group.

**Table 2:** LOD and LOQ values (n = 6) for both the FIA-MSX-MS/MS and UHPLC-MS/MS methods in corn.

Mycotoxin	FIA-MSX-MS/MS			UHPLC-MS/MS		MRL ^2^ (μg/kg)
	LOD (μg/kg)	LOQ (μg/kg)	Corr. LOQ (μg/kg) ^3^	LOD (μg/kg)	LOQ (μg/kg)	
AFB1	1.5	2.8	3.3	0.125 ^1^	0.2	5
AFB2	1.1	2.4	3.0	0.125 ^1^	0.3	5
AFG1	1.4	2.8	3.7	0.125 ^1^	0.2	5
AFG2	2.1	2.8	3.8	0.125 ^1^	0.3	5
DON	533	640	752.9	105	128	1750
FB1	14.4	20	28.2	22	30	1000
FB2	9.6	20	26.7	21	30	1000
HT2	43	64	68.8	42	61	100
OTA	2.4	3.5	4.7	0.3	1	5
T2	4.8	10	11.0	1.25	4	100
ZEA	41.6	64	73.6	1.75	4	200

^1^ Lowest point of the examined concentration range. ^2^ MRL refers to the unprocessed corn [21]. ^3^ LOQ corrected with the recoveries.

**Table 3:** LOD and LOQ values (n = 6) for both the FIA-MSX-MS/MS and UHPLC-MS/MS methods in wheat.

Mycotoxin	FIA-MSX-MS/MS			UHPLC-MS/MS		MRL ^2^ (μg/kg)
	LOD (μg/kg)	LOQ (μg/kg)	Corr. LOQ (μg/kg) ^3^	LOD (μg/kg)	LOQ (μg/kg)	
AFB1	1.05	1.5	1.8	0.125 ^1^	0.3	2
AFB2	0.4	1	1.2	0.15	0.5	2
AFG1	0.9	1.5	1.8	0.125 ^1^	0.3	2
AFG2	0.6	5	1.9	0.15	0.5	2
DON	355	480	619.2	280	320	1250
HT2	14.5	20	25.8	20	40	50
OTA	1.35	2	2.6	3.1	5	5
T2 toxin	11	15	17.0	8	16	50
ZEA	45.5	60	71.4	0.5	2.5	100

^1^ Lowest point of the examined concentration range. ^2^ MRL refers to the unprocessed wheat [21]. ^3^ LOQ corrected with the recoveries.

**Table 4:** Linearity and the dynamic range (n = 6) for both FIA-MSX-MS/MS and UHPLC-MS/MS in the examined matrices.

Mycotoxin	FIA-MSX-MS/MS				UHPLC-MS/MS			
	Corn		Wheat		Corn		Wheat	
	R^2^	Range (μg/kg)	R^2^	Range (μg/kg)	R^2^	Range (μg/kg)	R^2^	Range (μg/kg)
AFB1	0.9989	2.8–600	0.9977	1.5–350	0.9990	0.2–100	0.9988	0.3–100
AFB2	0.9956	2.4–600	0.9967	1–350	0.9981	0.3–100	0.9975	0.5–100
AFG1	0.9977	2.8–600	0.9983	1.5–350	0.9985	0.2–100	0.9981	0.3–100
AFG2	0.9973	2.8–600	0.9969	2–350	0.9982	0.3–100	0.9984	0.5–100
DON	0.9937	640–9600	0.9937	480–2400	0.9991	128–3500	0.9982	320–9600
FB1	0.9971	20–1200	- ^1^	- ^1^	0.9997	30–3500	- ^1^	- ^1^
FB2	0.9931	20–1500	- ^1^	- ^1^	0.9998	30–3500	- ^1^	- ^1^
HT2	0.9931	64–5200	0.9943	20–350	0.9993	61–3500	0.9978	40–240
OTA	0.9986	4.7–600	0.9973	2–350	0.9972	1–1000	0.9999	8–1400
T2	0.9986	10–800	0.9958	15–600	0.9985	4–250	0.9953	16–480
ZEA	0.9971	64–4800	0.9962	60–600	0.9981	4–500	0.9984	2.5–240

^1^ There are no MRLs for FB1 and FB2 mycotoxins in wheat; therefore, their parameters were not determined in this matrix.

**Table 5:** Matrix factors and their deviations (n = 12) in the examined matrices, determined for the FIA-MSX-MS/MS and UHPLC-MS/MS methods.

Mycotoxin	FIA-MSX-MS/MS				UHPLC-MS/MS			
	Corn		Wheat		Corn		Wheat	
	Matrix Factor	RSD%	Matrix Factor	RSD%	Matrix Factor	RSD%	Matrix Factor	RSD%
AFB1	0.25	17.1	0.14	14	0.78	5.0	0.68	7.6
AFB2	0.18	18.5	0.19	14.8	0.80	5.2	0.71	6.5
AFG1	0.14	18.5	0.16	12.2	0.81	11.5	0.69	7.1
AFG2	0.12	19.9	0.07	17.2	0.83	8.4	0.65	7.0
DON	0.28	17.3	0.28	18.6	0.64	13.8	0.68	14.2
FB1	0.51	14.6	- ^1^	- ^1^	0.78	8.6	- ^1^	- ^1^
FB2	0.96	9.4	- ^1^	- ^1^	0.81	13.7	- ^1^	- ^1^
HT2	0.35	14.7	0.79	14.4	0.72	6.8	0.79	9.2
OTA	1.11	8.4	0.92	14.6	0.74	6.9	0.86	8.7
T2	0.34	15.9	0.42	19.1	0.86	9.2	0.81	11.2
ZEA	0.64	15.6	1.21	9.3	0.80	7.8	0.79	8.9

^1^ There are no MRLs for the FB1 and FB2 mycotoxins in wheat; therefore, their parameters were not determined in this matrix.

**Table 6:** Recoveries and their deviations (n = 12) in the examined matrices determined with the FIA-MSX-MS/MS and UHPLC-MS/MS methods.

Mycotoxin	FIA-MSX-MS/MS				UHPLC-MS/MS			
	Corn		Wheat		Corn		Wheat	
	Recovery	RSD%	Recovery	RSD%	Recovery	RSD%	Recovery	RSD%
AFB1	0.86	13.8	0.78	9.2	0.87	12.7	0.81	11.13
AFB2	0.81	7.4	0.83	8.3	0.82	11.4	0.79	8.81
AFG1	0.76	6.8	0.79	8.6	0.86	9.9	0.88	13.81
AFG2	0.74	6.9	0.78	8.5	0.84	7.8	0.81	6.51
DON	0.85	4.9	0.71	11.8	0.82	6.8	0.79	9.15
FB1	0.71	12.1	- ^1^	- ^1^	0.79	7.9	- ^1^	- ^1^
FB2	0.75	7.9	- ^1^	- ^1^	0.76	8.7	- ^1^	- ^1^
HT2	0.93	6.1	0.71	11.3	0.88	11.8	0.81	9.15
OTA	0.74	7.2	0.71	8.4	0.81	6.8	0.76	7.88
T2	0.91	7.2	0.87	16.5	0.79	5.8	0.81	9.18
ZEA	0.87	8.2	0.81	11.4	0.91	6.8	0.87	12.1

^1^ There are no MRLs for the FB1 and FB2 mycotoxins in wheat; therefore, their parameters were not determined in this matrix.

## Data Availability

We have full control of all primary data, and we agree to allow the journal to review our data if requested.
